# Global Metabolic Regulation of the Snow Alga *Chlamydomonas nivalis* in Response to Nitrate or Phosphate Deprivation by a Metabolome Profile Analysis

**DOI:** 10.3390/ijms17050694

**Published:** 2016-05-10

**Authors:** Na Lu, Jun-Hui Chen, Dong Wei, Feng Chen, Gu Chen

**Affiliations:** 1 School of Food Science and Engineering, South China University of Technology, Guangzhou 510641, China; lvnahzau@gmail.com (N.L.); handcj@126.com (J.-H.C.); sfchencoe@pku.edu.cn (F.C.); chengu@scut.edu.cn (G.C.); 2 Institute for Food and Bioresource Engineering, College of Engineering, Peking University, Beijing 100871, China

**Keywords:** *Chlamydomonas nivalis*, nutrient deprivation, metabolome profile, responding biomarker, GC/TOF-MS, OPLS-DA

## Abstract

In the present work, *Chlamydomonas nivalis*, a model species of snow algae, was used to illustrate the metabolic regulation mechanism of microalgae under nutrient deprivation stress. The seed culture was inoculated into the medium without nitrate or phosphate to reveal the cell responses by a metabolome profile analysis using gas chromatography time-of-flight mass spectrometry (GC/TOF-MS). One hundred and seventy-one of the identified metabolites clustered into five groups by the orthogonal partial least squares discriminant analysis (OPLS-DA) model. Among them, thirty of the metabolites in the nitrate-deprived group and thirty-nine of the metabolites in the phosphate-deprived group were selected and identified as “responding biomarkers” by this metabolomic approach. A significant change in the abundance of biomarkers indicated that the enhanced biosynthesis of carbohydrates and fatty acids coupled with the decreased biosynthesis of amino acids, *N*-compounds and organic acids in all the stress groups. The up- or down-regulation of these biomarkers in the metabolic network provides new insights into the global metabolic regulation and internal relationships within amino acid and fatty acid synthesis, glycolysis, the tricarboxylic acid cycle (TCA) and the Calvin cycle in the snow alga under nitrate or phosphate deprivation stress.

## 1. Introduction

*Chlamydomonas nivalis*, a typical unicellular green microalga, is distributed worldwide in the snowfield of polar regions and similar extreme environments, and it has attracted attention recently due to its application as a model species of snow algae for illustrating the cellular response mechanism to stress conditions [1]. Nutrient deprivation is the most important stress factor that significantly impacts algal survival in harsh habitats, and it induces various cell responses through different mechanisms in the microalgae to adapt to the stress [2]. Of all the nutrients, nitrogen and phosphorus are two macronutrients with broad effects on algal growth and survival through complex biochemical processes, involving metabolic energy generation and transformation [3,4], membrane stability and signaling [5] and photosynthetic metabolism [6]. When a specific nutrient is deprived, the corresponding responses of the algal cells tend to regulate the metabolic pathways to compensate for the nutrient limitation [7]. For example, the rapid removal of nitrogen from the medium activates the assimilation of inorganic nitrogen, making nitrogen available through the production and re-use of NH_4_^+^ [8]. Phosphorus limitation leads to the up-regulation of protein digestion associated with lipid accumulation, while the down-regulation of protein biosynthesis is associated with amino acid metabolism in the marine diatom *Phaeodactylum tricornutum* [9]. The responses to sulfur starvation are quite similar to those of phosphorus starvation. The uptake and assimilations of cellular sulfate-containing metabolites are induced immediately, which directly affect the metabolism of vitamins and polyamines in *Arabidopsis* [10]. Most previous reports indicate that the responses of microalgae, corresponding to nutrient limitation as stress conditions, are achieved through various molecular regulations in different metabolic pathways.

The metabolome profile reflects the cellular metabolic stage at a specific physiological phase in response to environmental changes [11]. A metabolome profile analysis is a valid approach towards a quantitative description of the regulated and controllable process under stress conditions [12], and therefore, it provides unique perspectives of the fundamental regulations in algal cells. Gas chromatography time-of-flight mass spectrometry (GC/TOF-MS) is an effective method for metabolome profile analyses obtaining a global vision of the small molecules of metabolites (molecular weight <1000 dalton) due to its convenience and high accuracy in identifying and quantifying more than 100 metabolites in biological samples by a notably fast scan from a complicated matrix [13]. These small molecules, which include carbohydrates, organic acids, alcohols, aldehydes and amines, provide a comprehensive overview in the primary metabolic pathways [14]. The detailed procedures for metabolite profiling based on GC/TOF-MS have been established using *Chlamydomonas reinhardtii* as the model species [15,16,17,18], which are crucial for investigating the metabolism of microalgae. Based on these approaches and methods, the metabolic pathways of *Chlamydomonas reinhardtii*, changing accordingly under the deficiencies of nutrients (nitrogen, phosphorus, sulfur and iron), were investigated recently using GC/TOF-MS [19,20]. However, due to complications in establishing stable technical procedures of the sampling and extraction, only a few algal studies using GC-MS have been reported, which are worthy of further investigation to explore the algal metabolisms by GC/TOF-MS [15,21,22].

The Kyoto encyclopedia of genes and genomes (KEGG) [23] is an online tool to reconstruct the metabolic network from a similar organism and to manually draw pathway maps based on the representing knowledge about the molecular reactions, the regulation of metabolic pathways and the genomic information in various organisms. Pandhal *et al.* [24] investigated the metabolism of the cyanobacterium *Synechocystis* sp. PCC6803 (*Synechocystis* 6803) and its adaption to the high salinity stress by connecting the metabolomic and proteomic data to the KEGG pathway. The nitrogen-starved metabolism of the diatom was investigated by linking the change in the protein biosynthesis to the carbon metabolisms in the KEGG pathway [25].

In our previous studies, the responses of *C. nivalis* to salt stress and nutrient starvation (nitrate or phosphate deprivation stress) were investigated on a lipidomic level using UPLC/QTOF-MS (ultra-performance liquid chromatography/quadrupole time-of-flight mass spectrometry) coupled with OPLS-DA (the orthogonal partial least squares discriminant analysis), and more than twenty kinds of lipid/fatty acid biomarkers were selected and identified [5,26,27,28]. However, the biological functions of these biomarkers, involving membrane stability, signaling and photosynthesis, are under speculation according to the literature. It is difficult to link the changes of lipid biomarkers in the membrane system to the intercellular metabolic network. Therefore, the aim of this work is to elucidate the metabolic changes in certain stress conditions and to reveal the possible regulation mechanisms under these stress conditions by coupling GC/TOF-MS with a multivariate statistical analysis.

In the present study, GC/TOF-MS was first applied to detect the whole metabolome in the different stress groups of *C. nivalis* cells, and a multivariate statistical analysis (OPLS-DA) was employed to recognize patterns of the metabolome data from the groups. Then, the potential “responding biomarkers” were selected and identified. Significant changes in the abundance of the responding biomarkers were discussed subsequently, and finally they were visualized in the map of the KEGG pathway to reveal the global regulation of the metabolic network under these stress conditions.

## 2. Results and Discussion

### 2.1. Identification and Overview of the Metabolites

By rapid sampling, extraction and standard analysis using GC/TOF-MS, 670 metabolite peaks were detected and imported to the BinBase database, but only 171 metabolites were identified with a chemical structure and name. Overall, they belonged to several classes, including carbohydrates, organic acids, polyols, amino acids, *N*-compounds (nitrogen-containing compounds except amino acids), phosphates, fatty acids and other compounds.

**Figure 1:**
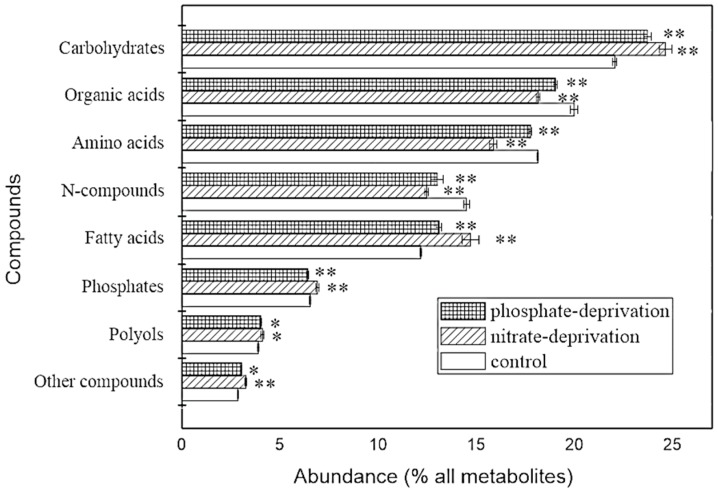
Overview of the identified metabolites in the classes and their abundance in the stress groups and the control group. *, ** mean significant difference (*p* < 0.05 or *p* < 0.01).

Figure 1 shows the overview of the identified metabolites in classes and their abundance in the stress groups and the control. The composition in the classes looks similar in all the groups, but the abundance of each class was significantly different compared to the control. Similar changes in the trends of their abundance in all of the classes were found in both of the stress groups; except that the class of phosphates increased significantly (*p* < 0.01) in the nitrate-deprived group but decreased significantly (*p* < 0.01) in the phosphate-deprived group.

Furthermore, comparing the abundance with that of the control, the carbohydrates significantly increased by 11.78% and 7.62% in the nitrate- and phosphate-deprived groups (*p* < 0.01), respectively. This could be due to the decreased catabolism and reutilization of the stored carbon compounds for the biosynthesis under both stress conditions [29]. Amino acids decreased by 12.47% and 2.09% (*p* < 0.01) in both of the stress conditions, and *N*-compounds also decreased significantly by 14.06% and 10.35%, respectively (*p* < 0.01). Protein biosynthesis is slowed down by low nitrate and phosphate concentrations [29]. A nitrogen limitation reduces the protein synthesis, mainly due to the limited translation of mRNA without the efficient supply of amino acids, while a phosphorus limitation causes a short supply of adenosine triphosphate (ATP), which leads to the accumulation of carbohydrates in preference to proteins due to the fact that the enzymes for the protein biosynthesis have a low affinity for ATP [30]. Additionally, organic acids, including the central intermediates from glycolysis, the citric acid cycle and pyruvate metabolism, decreased by 9.2% and 4.8%, respectively (*p* < 0.01). However, fatty acids increased 20.9% and 7.65%, respectively (*p* < 0.01). Excess carbon resources are preferred to biosynthesize fatty acids during nutrient limitation, which can explain the increased triglyceride in response to the nitrogen or phosphate limitation [25]. Therefore, following evidence of a global metabolic rebalance in the stress groups, the investigation in this work was extended toward a metabolome profile analysis.

### 2.2. Discrimination of Metabolomic Data by OPLS-DA (the Orthogonal Partial Least Squares Discriminant Analysis)

**Figure 2:**
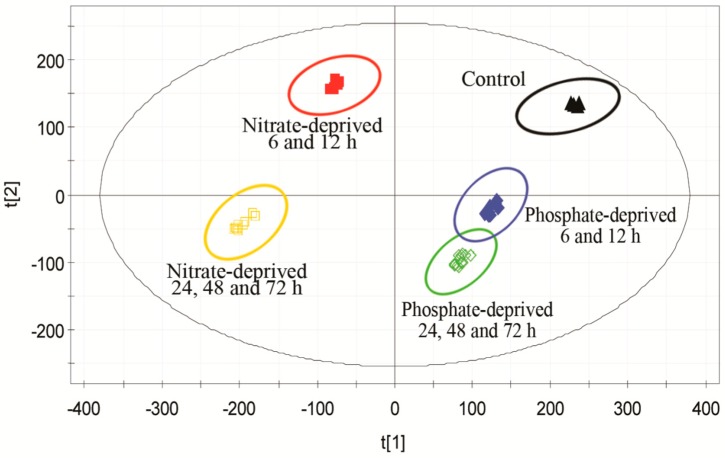
Score scatter plot generated by the orthogonal partial least squares discriminant analysis (OPLS-DA) model from the peak area of the identified metabolites in the different groups. (▲) the control; (■) nitrate deprivation for 6 and 12 h; (□) nitrate deprivation for 24, 48 and 72 h; (◆) phosphate deprivation for 6 and 12 h; (◇) phosphate deprivation for 24, 48 and 72 h. t[1], the score of the first predictive component to explain the largest variation; t[2], the score of the first orthogonal component to explain the largest orthogonal variation.

To assess the differentiation between the stress groups and the control on a metabolomic level, the OPLS-DA model was employed to discriminate the origins of the metabolite profiles [17]. The score scatter plot in Figure 2 was generated from the peak area of the identified metabolites in the different groups. Five experimental clusters with time points were clearly separated by the OPLS-DA model, where each spot represents a sample with the parameters of *R*^2^*X*, *R*^2^*Y*, and *Q*^2^ (0.944, 0.981, and 0.971, respectively). The samples from the different experimental groups were analyzed repeatedly to validate the original model, which showed that this procedure demonstrated great repeatability from the stress treatments to the data analysis. In the “*y*-scrambling” validation that was applied for this OPLS-DA model, random permutation tests were performed, and the parameter of *Q*^2^ (−0.364 < 0) indicated that the valid model had high predictability without overfitting [31].

In Figure 2, the samples that were stressed for 6 and 12 h clustered together in the nutrient-deprived groups and separated with another cluster (stressed for 24, 48, and 72 h), indicating that 6 and 24 h might be the turning point of this alga for the essential metabolic regulation responding to the nutrient deprivation stress. All the clusters in the phosphate-deprived group located closer to the control, and thus, their metabolite profiles are more similar to the control than the nitrate-deprived group. This could be due to the fact that the algae were not yet fully P-deprived at the beginning of the P-stressed treatment. The organisms often store substantial amounts of excess P as polyphosphates to maintain the phosphorus use efficiency during P deprivation [32]. These results also indicated that the metabolism in this alga was affected more effectively by nitrate than phosphate, and the OPLS-DA model was perfect for obtaining metabolic readouts in investigating the influence of nutrients in the culture of *C. nivalis*.

### 2.3. Selection of the Responding Biomarkers

**Figure 3:**
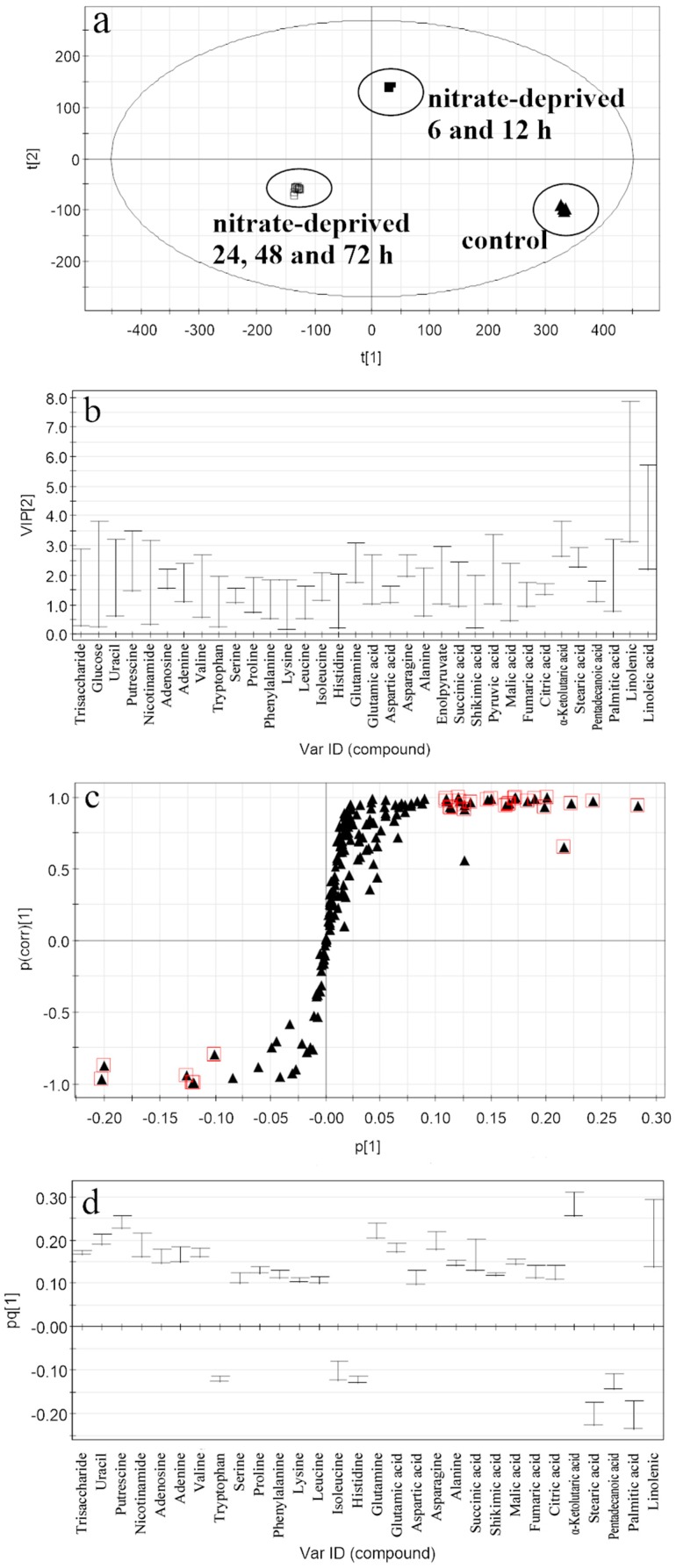
Related plots of the OPLS-DA model between the nitrate-deprived group and the control. (**a**) Score scatter plot for metabolite discrimination. (▲) the control; (■) nitrate-deprived group for 6 and 12 h; (□) nitrate-deprived group for 24, 48 and 72 h; (**b**) VIP plot (with VIP > 1 metabolites); (**c**) s-plot; square marked metabolites are those with |*p*(corr)| > 0.6; (**d**) Loading column plot of the potential metabolites biomarkers.

**Figure 4:**
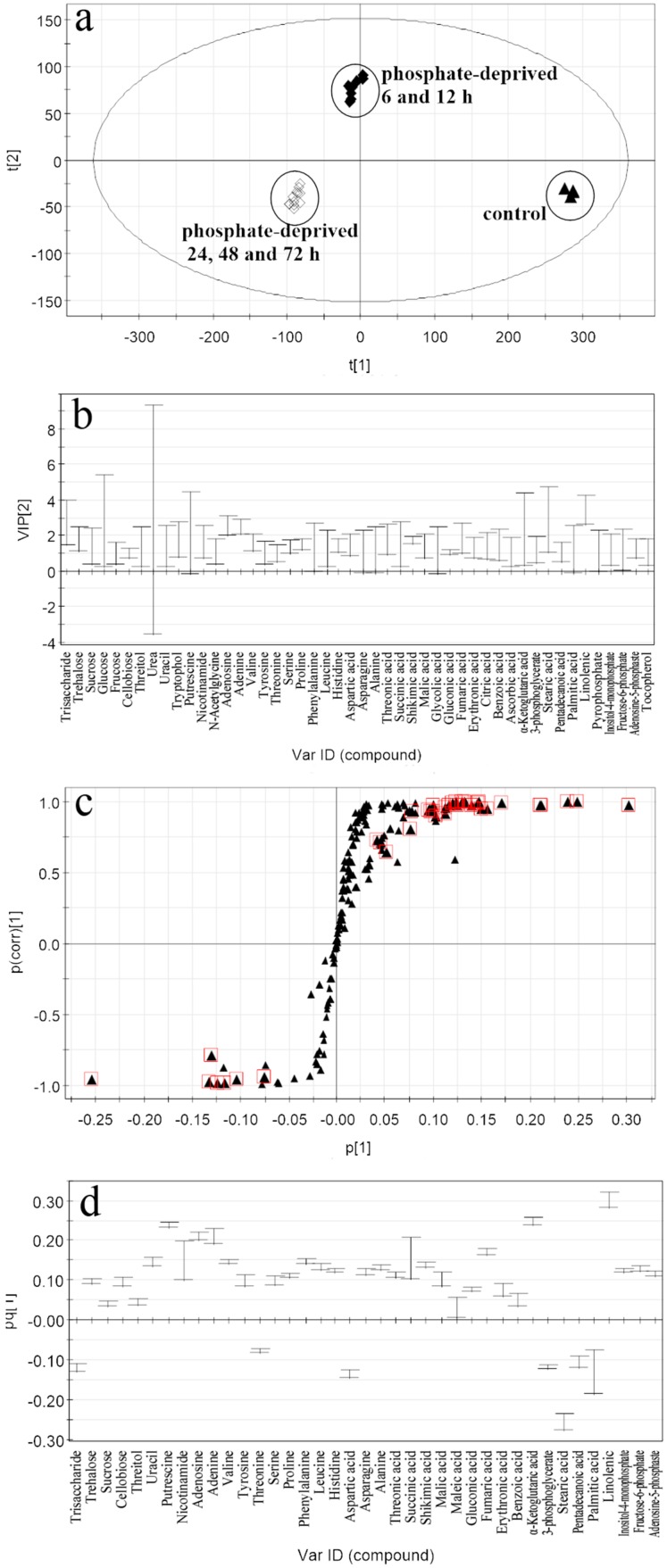
Related plots of the OPLS-DA model between the phosphate-deprived group and the control. (**a**) Score scatter plot for metabolite discrimination. (▲) the control; (◆) phosphate-deprived group for 6 and 12 h; (◇) phosphate-deprived group for 24, 48 and 72 h; (**b**) VIP plot (with VIP > 1 metabolites); (**c**) s-plot; square marked metabolites are those with |*p*(corr)| > 0.6; (**d**) Loading column plot of the potential metabolites biomarkers.

To illustrate the pattern of metabolic regulation that responds to nutrient-deprived stress in *C. nivalis*, key contributors among the metabolites that clustered needed to be identified as responding biomarkers. For this purpose, the related plots of the OPLS-DA models shown in Figure 3 and Figure 4 were generated from both of the stress groups *vs.* the control, respectively. The performance of *R*^2^*X*, *R*^2^*Y*, and *Q*^2^ in the OPLS-DA model generated from the nitrated-deprived group *vs.* the control (Figure 3a) was 0.953, 0.999, 0.998, respectively. The intercept value of *Q*^2^ in the permutation test was −0.34. All the results showed that the established model is robust and not overfitting. The procedure for the selection of the responding biomarkers was the same as described in our previous work [28]. Briefly, the potential biomarkers had to meet all of the three criteria (*i.e.*, VIP (variable influence on the projection) > 1, ｜*p* (corr(correlation coefficient))｜> 0.6 and the span of CIJK_JK_(jack-knifed confidence interval) excluding 0) for further investigation. Overall, thirty metabolites were selected as “responding biomarkers” among which the largest VIP value was 5.48 from the nitrate-deprived group *vs.* the control. These included one carbohydrate, five *N*-compounds, fourteen amino acids, six organic acids and four fatty acids (shown in Table 1).

The performance of *R*^2^*X*, *R*^2^*Y*, and *Q*^2^ in the OPLS-DA model generated from the phosphate-deprived group *vs.* the control (Figure 4a) was 0.877, 0.987, 0.971, respectively, and the intercept value of *Q*^2^ in the permutation test was −0.348, indicating that the established model is also robust and not overfitting. According to the same procedure above, thirty-nine of the identified metabolites were selected as “responding biomarkers” among which the largest VIP value is 3.42 from the phosphate-deprived group *vs.* the control. These included five carbohydrates, four *N*-compounds, twelve amino acids, ten organic acids and eight fatty acids (shown in Table 2).

Based on the same criteria, the results showed that less responding biomarkers were selected from the nitrate-deprived group, but the largest VIP value was higher than that from the phosphate-deprived group, indicating that nitrate has a greater effect than the phosphate on the metabolomic profiling. Especially, the unique biomarkers discovered only from the nitrate-deprived group *vs.* the control were tryptophan, lysine, isoleucine, glutamine and citric acid. Most of these are amino acids (in Table 1), suggesting that nitrate deprivation has a greater influence on amino acid metabolism. While, the unique biomarkers discovered only from the phosphate-deprived group *vs.* the control were trehalose, sucrose, cellobiose, threitol, tyrosine, threonine, threonic acid, gluconic acid, erythronic acid lactone, benzoic acid, 3-phosphoglycerate, pyrophosphate, inositol-4-monophosphate, fructose-6-phosphate and adenosine-5-phosphate. Most of these are carbohydrates and organic acids related to central carbon metabolism (Table 2).

### 2.4. Abundance of the Responding Biomarkers in the Different Groups

**Figure 5:**
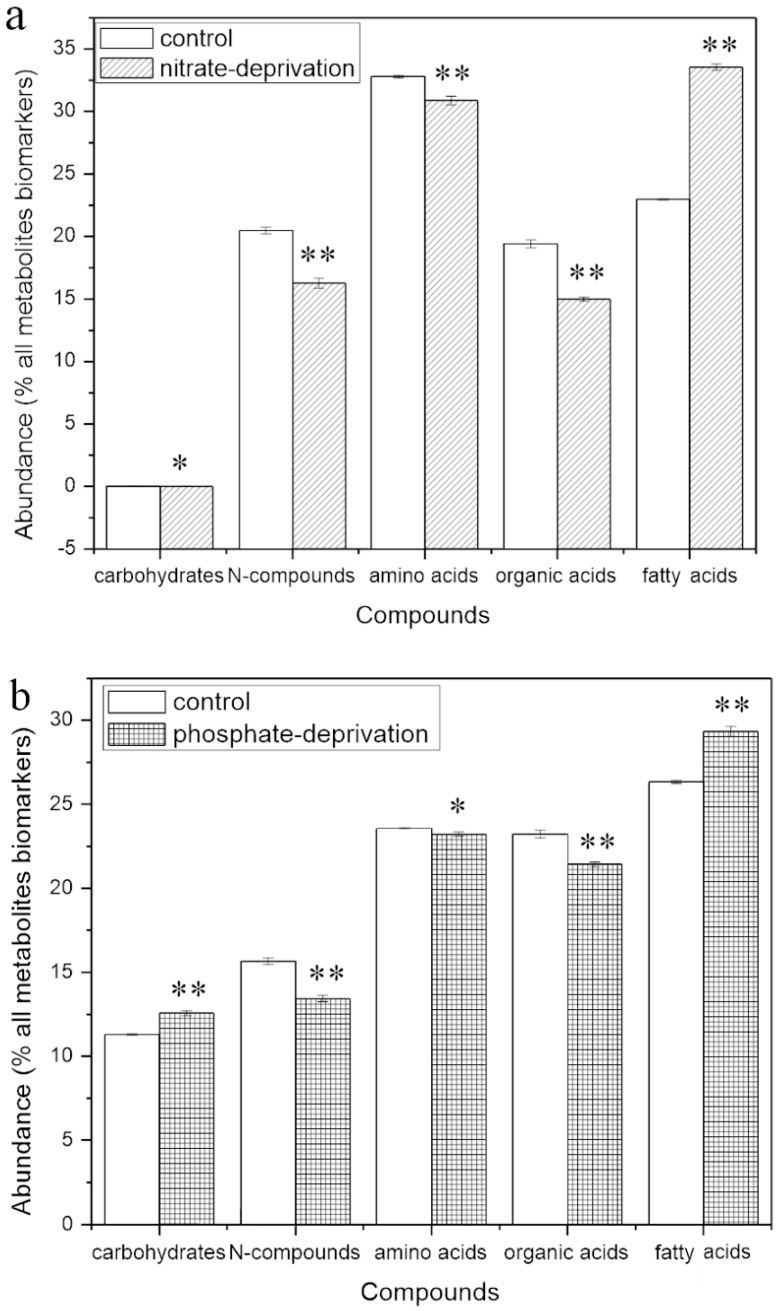
The abundance in the biomarker classes responding to the stresses compared with the control*.* *, ** mean significant difference (*p* < 0.05 or *p* < 0.01).

The abundance of the responding biomarkers in the classes in the different groups is shown in Figure 5. Although the abundance changes in all the classes were significantly different compared to the control, the changes in the biomarker classes have the same trends as that of the whole identified metabolite classes as shown in Figure 1. Therefore, the cellular response and adaptation to both nutrient deprivations could be represented by the changes in the abundance in the responding biomarkers during the certain stress.

As shown in Figure 5, the abundance of the carbohydrate biomarkers significantly increased by 11.46% (*p* < 0.01, Figure 5a), which was mainly trisaccharide (Table 1), and by 31.34% (*p* < 0.05, Figure 5b), which was mainly trisaccharide, trehalose, sucrose and cellobiose (Table 2), in the N and P stressed groups. However, a significant decrease in the abundance of the *N*-compound (*p* < 0.01) and amino acid (*p* < 0.05) biomarkers was found in the two stress groups. Moreover, the decrease in the abundance of the amino acid biomarkers in the N deprivation group (*p* < 0.01) was much higher than in the P deprivation group (*p* < 0.05), indicating that N deprivation affects the metabolism of amino acids more effectively. The abundance of the fatty acid biomarkers significantly increased by 46.12% (*p* < 0.01, Figure 5a) and 11.45% (*p* < 0.01, Figure 5b) in the two stress groups, which is in accordance with that of the carbohydrate biomarkers. Linolenic acid (18:3), stearic acid (C18:0), palmitic acid (16:0), and pentadecanoic acid (15:0) were the fatty acid biomarkers with the largest variation in both of the stress groups and also increased in all the nitrate-deprived groups (Table 1), but the linolenic acid decrease was observed only in the phosphate-deprived group (Table 2).

It was proposed that changes in carbon and nitrogen metabolism to regulate their balance for storing excess carbon in molecular pools are crucial under conditions of limited or no nitrogen. When higher amounts of polysaccharides are released, this was caused by a switch in metabolism from protein to carbohydrate [29]. However, the metabolic pathways and the enzymes activated by the nutrient deprivations were quite different in various microalgae. For example, the overexpression of acetyl-CoA carboxylase, which increases oil production in green algae, does not increase oil accumulation in diatom [33]. Although nitrogen affects the biosynthesis of fatty acids and lipids, the direct explanation for the effects in algae is not clearly understood at this time. The assimilation of carbon under nitrogen starvation is tuned to accumulate carbon skeletons and reduce equivalents for the fatty acid biosynthesis instead of nitrogenous compounds, such as amino acids [25]. In the freshwater green alga *Chlamydomonas reinhardtii*, excess carbon is directed to the biosynthesis of fatty acids and lipids under nitrogen-deprived conditions [34] and the up-regulation of the expression of the related genes [35].

#### 2.4.1. Responding Biomarkers in the Nitrate-Deprived Group

In the nitrate-deprived group, five *N*-compounds and fourteen amino acids were selected as responding biomarkers, and the change rates in abundance are shown in Table 1. Among these, eleven amino acid biomarkers (valine, serine, proline, phenylalanine, lysine, leucine, glutamine, glutamic acids, aspartic acid, asparagine and alanine) decreased from 1.03% to 58.9%, contributing to the most declines of the total amino acids. Asn, Asp, Pro and Gln are well known to be involved in the biosynthesis of nitrogen compounds and the metabolism of nitrogen transportation. Reducing the supply of these amino acids suddenly led to the reduction of the de novo biosynthesis of several amino acids. Conversely, three amino acid biomarkers (tryptophan, isoleucine and histidine) increased by 85.84%, 75.56% and 86.94%, respectively. This might be due to the fact that the enzymes that enhance the nitrogen uptake were induced in response to N deprivation, while the other protein biosynthesis was reduced due to the decreased availability of the nitrogen sources [35]. These results indicate that *C. nivalis* is incapable of maintaining the homeostasis of amino acid metabolism *in vivo* under this stress due to changes in the biosynthesis of several amino acids and amino group mediators.

The ratio of glutamic acid to glutamine (Glu/Gln) decreased 19.69% (from 0.52 to 0.42), while the proportion of glutamine and α-ketoglutaric acid increased 68.94% (from 0.96 to 1.63) in the nitrate-deprived group (Table 1). The proportion of glutamine/α-ketoglutaric acid is crucial to affect the bioactivities of glutamine synthetase and glutamate synthase under nitrogen stress, and it remains stable by regulating the capability of utilizing alternative nitrogen sources in several metabolisms [36]. Specially, the increase of this ratio (glutamine/α-ketoglutaric acid) leads to a decreased capability of nitrogen transport and metabolism and protein turnover (N-recycling) and an increased mobilization of C-reserves. In addition, citric acid, α-ketoglutaric acid and the downstream metabolites (fumaric acid and malic acid) in the tricarboxylic acid cycle (TCA) decreased 33.14%, 55.11%, 34.74% and 11.30%, respectively in the nitrate-deprived group (Table 1). These decrease indicated that an inhibition effect occurred in the TCA cycle in the N-stress condition [37]. However, a small increase of 8.36% for succinic acid was found in our experiments as shown in Table 1. As discussed before, reduced nitrate assimilation leads to the accumulation of reducing equivalents (NADH), and thus, less malic acid is formed from succinic acid.

#### 2.4.2. Responding Biomarkers in the Phosphate-Deprived Group

The declines of 43.47% for intracellular adenosine and 4.98% for adenosine-5-phosphate (AMP) were the distinct metabolic changes observed in response to phosphate deprivation (Table 2). The same trend was found in *Selenastrum minutum* [38]. Because the activity of certain glycolytic enzymes was affected by the supplementation of ATP, ADP (adenosine diphosphate) or Pi (inorganic phosphate), the reduced biosynthesis of these metabolites had a great impact on the function of glycolysis in the algal cells. The reduced respiration rates and the consequential reduction in the Calvin cycle and light utilization were also observed in *Selenastrum* sp. due to the cellular metabolite changes under a phosphorus limitation [39].

Inositol-4-monophosphate decreased 37.26% due to the Pi limitation (Table 2). Inositol-4-monophosphate is a phosphorylated inositol and plays key roles in cellular metabolic processes, especially those involved in the signaling pathway of phosphophatidylinositol. Thus, it could partly explain the decreased cell growth of *C. nivalis* in the phosphate-deprived stress condition [5]. Interestingly, pyrophosphate increased 41.9% in this stress group. In general, pyrophosphate is presumably used to regulate the concentration of Pi in the cytoplasm, and it may play an important role in the storage of internal calcium and calcium mediated signal transduction [40]. Polyphosphate is mainly biosynthesized from the assimilation of the inorganic phosphate in the cells of *Chlamydomonas* under phosphate stress [41]. Park *et al.* [8] reported that high-phosphorus treatments induced a higher cellular consumption of phosphorus, which led to a significant increase of pyrophosphate in algae, but the treatments did not significantly affect the biosynthesis of polyphosphates, usually as the subsequent intracellular storage of phosphorus. The large accumulation of polyphosphates and pyrophosphate is also toxic to the microalgal cells because it depresses the key metabolite reactions related to proteins, nucleic acids and lipids [42]. Additionally, aspartic acid increased 42.85% in the phosphate-deprived group. Aspartic acid is an inhibitor of pyruvate carboxylase, which catalyzes the carboxylation of pyruvate to oxaloacetate in order to supply the intermediates of the citrate cycle [43]. Therefore, we speculated that the aspartic acid increase led to the inhibition of the activities of pyruvate carboxylase, which further demonstrated an inhibitory effect on the TCA cycle. These data are consistent with a previous study that aspartate inhibits pyruvate carboxylase competitively with respect to acetyl-CoA and decreases the levels of the TCA cycle intermediates [44]. Based on the above results, phosphorus deprivation increases the intermediates of the TCA cycle and reduces the substrates in the Calvin cycle and respiration.

### 2.5. Metabolic Pathway and Regulation Analysis under Stresses

**Figure 6:**
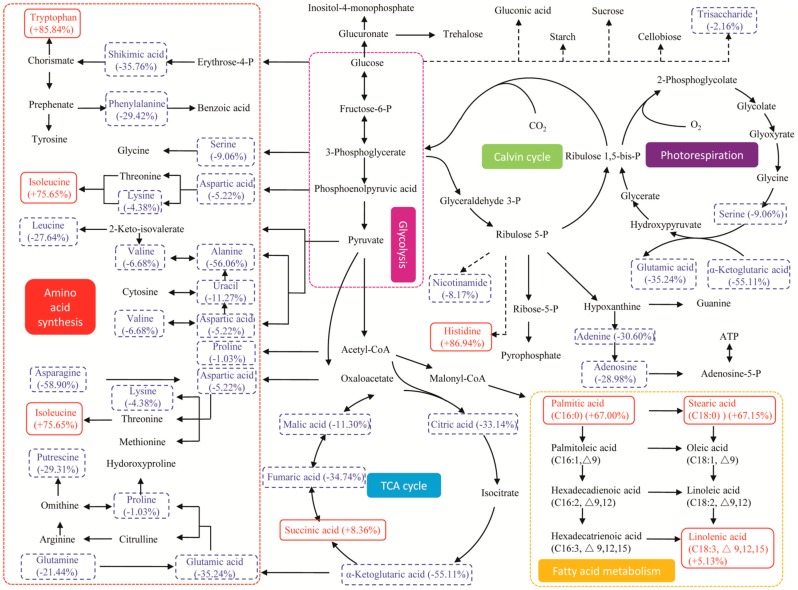
Metabolic pathway and regulation visualized by Kyoto encyclopedia of genes and genomes (KEGG) under nitrate-deprived stress. Direct connections between the metabolites are indicated with solid line arrows, and putative connections between the metabolites are indicated with dash line arrows. The red words in red solid line boxes represent an increase in the abundance of the responding biomarkers, the blue words in blue dash line boxes represent a decrease in the abundance, and the black words represent no significant change. The percentages represent the change rates of the biomarkers by up- or down-regulation.

**Figure 7:**
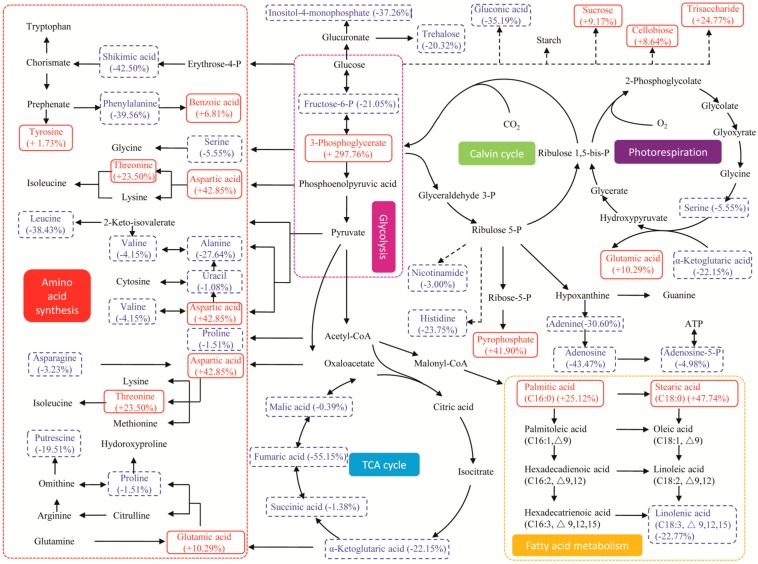
Metabolic pathway and regulation visualized by KEGG under phosphate-deprived stress. Direct connections between the metabolites are indicated with solid line arrows, and putative connections between the metabolites are indicated with dash line arrows. The red words in red solid line boxes represent an increase in the abundance of the responding biomarkers, the blue words in blue dash line boxes represent a decrease in the abundance, and the black words represent no significant change. The percentages represent the change rates of the responding biomarkers by up- or down-regulation.

To visualize the global regulation of the metabolic network in which the responding biomarkers are involved, the IDs (Identity) of the biomarkers in the Binbase database were matched with the IDs in KEGG and were then mapped on the metabolic pathways based on the *Chlamydomonas reinhardtii* pathway network in the KEGG database [45]. Almost all the responding biomarkers (threonic acid, threitol, erythronic acid-1,4-lactone and pentadecanoic acid were not found in the KEGG pathways) in the major pathways and their regulation referred as the change rates in abundance under stresses are shown in Figure 6 and Figure 7. Seven central pathways in microalgae are included, such as photorespiration, the Calvin cycle, glycolysis, amino acid synthesis, the TCA cycle, fatty acid metabolism, succinate production and glutamate synthesis.

Two major amino acids (serine and glutamic acid) and α-ketoglutaric acid were identified as biomarkers participating in photorespiration (Figure 6 and Figure 7). In the nitrate-deprived group, a decline of their abundance indicated a decrease in photorespiration (Table 1); while, in the phosphate-deprived group, glutamic acid increased 10.29% and 3-phosphoglycerate increased 297.76% (Table 2), which could be synthesized through the enhanced photorespiration, the Calvin cycle and glycolysis for carbon cycling.

Three fatty acid biomarkers (linolenic acid, stearic acid and palmitic acid) participate in fatty acid metabolism. Their abundance changes showed the same trends in the two stress groups (Figure 6 and Figure 7) with increased contents of palmitic acid and stearic acid and a decreased content of linolenic acid, leading to the reduced abundance of unsaturated lipids [17] to enhance the stress tolerance of nutrient deprivation by changing the membrane fluidity and permeability [27]. The results are in accordance with that fact that the fatty acid biomarkers increased significantly in the two stress groups as described in Figure 5.

The metabolisms of several amino acid biomarkers are relatively complicated under different stress conditions. In the nitrate-deprived group (Figure 6), all the amino acid biomarkers mapped on the network were decreased except for isoleucine [34]. In the phosphate-deprived group (Figure 7), some of the amino acid biomarkers (aspartic acid, threonine, glutamic acid and tyrosine) increased, while others decreased, and thus, the regulatory mechanisms were not obvious. Five organic acids (succinic acid, citric acid, fumaric acid, malic acid, and α-ketoglutaric acid) were detected as responding biomarkers in the TCA cycle. In the nitrate-deprived group (Figure 6), succinic acid increased slightly at 8.36%, but this small increase cannot counteract the decline of the other organic acid biomarkers (Table 1). While in the phosphate-deprived group (Figure 7) all the organic acid biomarkers decreased, leading to a down-regulation of TCA activity.

Compared with the widely studied model strain, *Chlamydomonas reinhardtii*, the metabolic responses of *C. nivalis* under nutrient stresses were not well known. *C. nivalis* was originated from polar region and similar extreme environments, which were different from that of *C. reinhardtii*. It was reported that the snow alga *Chlamydomonas nivalis* is cryotolerant in comparison with the mesophilic *C. reinhardtii*, and it has the special mechanisms for adapting photosynthesis to low temperatures and changing thylakoid lipid composition to enhance the membrane fluidity [46]. The metabolic responses of *C. nivalis* to nitrogen and phosphate stresses in our study were investigated and compared with that of *C. reinhardtii* in previous studies. In our case, the metabolic pathways of nitrogen uptake and lipid biosynthesis were enhanced significantly in *C. nivalis* under nitrogen deprivation. The increased mobilization of C-reservers and the accumulation of reducing equivalents for the fatty acid biosynthesis were also observed in our study. These were similar with that of *C. reinhardtii* [6,8]. However, it’s worth to note that, for example, the arginine synthesis pathway in *C. reinhardtii* was up-regulated significantly after nitrogen deprivation in order to take exogenous available nitrogen rapidly [8]. This phenomenon was not found in *C. nivalis* in our study, which indicated the different regulation of the nitrogen uptake between the two alga species.

In contrast to nitrogen deprivation, phosphate deprivation has more effects on central carbon and energy metabolism in *C. nivalis*. Previous studies have shown that under phosphate starvation conditions, *C. reinhardtii* has evolved the adaptive mechanisms to recycle phosphate from the intracellular stocks, such as polyphosphate, pyrophosphate and other metabolites containing phosphate [47]. The increasing content of pyrophosphate was also found in *C. nivalis* under phosphate deprivation; however, the large accumulation of these metabolites depressed the cell growth to some extent. Phosphate deprivation resulted in the decrease of the organic acid biomarkers which affected TCA cycle in *C. nivalis*. Interestingly, it was found in plants that the release of organic acid is helpful for mobilizing phosphate and increasing its concentrations in soil solution [48]. As to the metabolism of amino acids, the distinct difference between the two algae was that cysteine and its pool were significantly increased in *C. reinhardtii* under phosphate deprivation [49] but not in *C. nivalis* in this study. Cysteine and its derivatives were the major metabolites of sulfur assimilation in *C. reinhardtii* [47]. The continual supply of sulfur is necessary to cellular functions and metabolic responses to nutrient-deprived stress. These results indicated sulfur metabolisms in the two algae might be different, which were worthy of further investigating. Therefore, it could be concluded that our work provides new insight for a more fundamental understanding of the metabolome and the regulation of metabolism in *Chlamydomonas nivalis*.

Overall, the change rates in the abundance of the responding biomarkers only represent the instantaneous state fixed in multiple metabolic pathways at the same time point. The regulatory strategy in each pathway becomes extremely complex by connecting it to a global metabolic network. By determining the limited responding biomarkers under stresses, it was very feasible to realize the visualization of the metabolic network. However, there are still some limitations on the interpretation due to the complications of the metabolism. In general, the metabolites are not only involved in multiple pathways, but are also presented in different subcellular pools. Another technical factor is that the current system does not offer enough precision to detect all the metabolites and/or to identify all the detected metabolites. Despite that, the metabolic pathway analysis and reconstruction, based on the responding biomarkers in the metabolomic analysis, is still a very useful tool for providing substantial information to elucidate the key regulatory points in the network and to reveal the global regulation model in microalgae under environmental stress.

## 3. Experimental Section

### 3.1. Materials

The snow alga *Chlamydomonas nivalis* LB-2824 were purchased from the Culture Collection of Algae at the University of Texas at Austin (UTEX), Austin, TX, USA. HPLC-grade methanol and chloroform were purchased from Merck Co. (Darmstadt, Germany), and the other analytical-grade chemicals and reagents were purchased from Jinke (Shanghai, China) Co., Ltd. Deionized water was obtained by using a Milli-Q water system (Millipore, Bedford, MA, USA). Methoxyamine hydrochloride, *N*-Methyl-*N*-(trimethylsilyl) trifluoroacetamide (MSTFA) and pyridine were purchased from Sigma-Aldrich (St. Louis, MO, USA).

### 3.2. Algal Culture, Stress Treatment and Biomass Harvest

The seed culture procedure, the nitrate- or phosphate-deprived stress treatment and the biomass harvest are reported in our previous work [5]. All the cultures were performed in triplicate for the experiments.

### 3.3. Extraction and Derivatization of the Cellular Compounds

Four milligrams of lyophilized algal powder and 1 mL of solvent (methanol:chloroform:water, 10:3:1) were mixed to extract the cellular compounds [50]. The extracts were derivatized by adding 10 µL of a solution of methoxyamine hydrochloride in pyridine (40 mg/mL) followed by shaking for 90 min at low temperature (30 °C) to prevent damage to the chemical groups (aldehyde and ketone). Sequentially, MSTFA (100 µL) was added into each reaction, and it was shaken at 37 °C for 30 min. As a negative control, one blank group without algal powder was performed in the whole procedure.

### 3.4. Metabolome Profile Analyses by Gas Chromatography Time-of-Flight Mass Spectrometry (GC/TOF-MS)

#### 3.4.1. Gas Chromatography Procedure

Agilent 6890 gas chromatography (Agilent, Santa Clara, CA, USA) equipped with a Gerstel CIS4-dual MPS Injector (Gerstel, Muehlheim, Germany) was employed for the metabolome profile analysis. Aliquot of the samples (0.5-µL) were splitless injected at 10 µL/s into Rtx-5 Sil MS column (30 m × 250 µm, 95% dimethyl and 5% diphenyl polysiloxane, 0.25 µm; Restek, Bellefonte, PA, USA). The injector temperature was raised from 50 to 275 °C by 12 °C/s and was held for 3 min. The temperature of the oven was held constant at 50 °C for 1 min, was raised to 330 °C by 20 °C/min and was then held for 5 min. The carrier gas was 99.9999% pure helium at a constant flow rate of 1 mL/min.

#### 3.4.2. Mass Spectrometry

A Leco Pegasus III time-of-flight mass spectrometer (LECO Corporation, St. Joseph, MI, USA) was used to analyze the metabolome profile. The shift temperature was set at 280 °C, and the ion source temperature was set to 250 °C in order to achieve the electron impact ionization at 70 eV. The acquisition rate was 17 spectra/s, and the scan mass ranged from 85 to 500 Da.

#### 3.4.3. Data Processing and Normalization

All the peaks of the detected metabolites were analyzed by Leco ChromaTOF software V2.32 (LECO Corporation, St. Joseph, MI, USA) followed by processing with the programmed database BinBase according to a former report [51]. The metabolite data were normalized according to our previous study [5]. Briefly, the ion intensities of each peak detected were normalized to the sum of the peak intensities and were then normalized to the cell number in the sample. The normalized data were multiplied by 10,000 and were transformed to log_10_ for establishing a normal distribution in each sample for further multivariate statistical analyses.

### 3.5. Multivariate Statistical Analyses

SIMCA-P+ V12.0 software (Umetrics AB, Umea, Sweden) was used to perform the OPLS-DA model. Pareto scaling was first applied to pretreat the data sets to make them more comparable in magnitude for the analysis. OPLS-DA is a useful statistical procedure to discriminate the different classes by comparing and selecting the informative variables, which adopt to class membership [5]. Permutation tests with 200 iterations were performed to evaluate the possible over-fitting of data. *R*^2^*X*, *R*^2^*Y* and *Q*^2^, varied between 0 and 1, are the primary parameters of the model, which indicates the fitting degree of the data. An intercept value of *Q*^2^ > 0.05 means that overfitting in the original model exists [31]. Potential responding biomarkers were identified based on the S-plot, the column loading plot and the VIP value [26,27,28]. The change rate in the percentage of certain biomarkers was calculated by comparing the abundance of the corresponding metabolites in the stress groups with the control. The significance test for the variation of the biomarker’s abundance was implemented by a student’s *t*-test.

## Figures and Tables

**Table 1:** Thirty of the selected metabolites with a VIP > 1 and a |*p* (corr)| > 0.6 as responding biomarkers from the nitrate-deprived group *vs.* the control group.

Number	Identified Metabolites	VIP	*p*[1]	*p*(corr)[1]	Change Rate in Abundance, % (N Deprivation *vs.* the Control)
1	Linolenic acid	5.48	0.22	0.65	5.13
2	α-Ketoglutaric acid	3.24	0.28	0.94	−55.11
3	Stearic acid	2.60	−0.20	−0.88	67.15
4	Putrescine	2.48	0.24	0.97	−29.31
5	Glutamine	2.42	0.22	0.95	−21.44
6	Asparagine	2.33	0.20	0.93	−58.90
7	Palmitic acid	2.01	−0.20	−0.97	67.00
8	Uracil	1.92	0.20	0.99	−11.27
9	Adenosine	1.87	0.16	0.93	−28.98
10	Glutamic acid	1.86	0.18	0.97	−35.24
11	Nicotinamide	1.75	0.19	0.99	−8.17
12	Adenine	1.74	0.17	0.96	−30.60
13	Succinic acid	1.71	0.17	0.94	8.36
14	Valine	1.65	0.17	0.99	−6.68
15	Isoleucine	1.61	−0.10	−0.80	75.65
16	Trisaccharide	1.59	0.17	1.00	−2.16
17	Citric acid	1.52	0.13	0.91	−33.14
18	Pentadecanoic acid	1.45	−0.13	−0.95	75.54
19	Alanine	1.43	0.15	0.98	−56.06
20	Malic acid	1.41	0.15	0.99	−11.30
21	Fumaric acid	1.36	0.13	0.94	−34.74
22	Aspartic acid	1.35	0.11	0.92	−5.22
23	Proline	1.34	0.13	0.97	−1.03
24	Serine	1.30	0.11	0.93	−9.06
25	Phenylalanine	1.19	0.12	0.97	−29.42
26	Histidine	1.13	−0.12	−0.99	86.94
27	Tryptophan	1.11	−0.12	−1.00	85.84
28	Shikimic acid	1.11	0.12	1.00	−35.76
29	Leucine	1.09	0.11	0.97	−27.64
30	Lysine	1.01	0.11	0.99	−4.38

VIP: variable influence on the projection; *p*[1]: the loading of the first predictive component to explain the largest variation; *p*(corr)[1]: the correlation coefficient between the metabolite and the score of the first predictive component to explain the largest variation; N: nitrogen.

**Table 2:** Thirty-nine of the selected metabolites with a VIP > 1 and a |*p*(corr)| > 0.6 as responding biomarkers from the phosphate-deprived group *vs.* the control group.

Number	Identified Metabolites	VIP	*p*[1]	*p*(corr)[1]	Change Rate in Abundance, % (P deprivation *vs.* the Control)
1	Linolenic acid	3.42	0.30	0.98	−22.77
2	Stearic acid	2.89	−0.25	−0.96	47.74
3	Trisaccharide	2.75	−0.12	−0.88	24.77
4	Adenosine	2.53	0.21	0.98	−43.47
5	Glutamic acid	2.49	0.21	0.97	10.29
6	α-Ketoglutaric acid	2.34	0.25	0.99	−22.15
7	Putrescine	2.16	0.24	1.00	−19.51
8	Fumaric acid	1.84	0.17	0.99	−55.15
9	Trehalose	1.81	0.10	0.93	−20.32
10	Threonic acid	1.79	0.11	0.92	1.05
11	Shikimic acid	1.75	0.14	0.97	−42.50
12	Nicotinamide	1.63	0.15	0.94	−3.00
13	Valine	1.60	0.15	0.98	−4.15
14	Succinic acid	1.51	0.16	0.94	−1.38
15	Proline	1.49	0.11	0.95	−1.51
16	Aspartic acid	1.49	−0.13	−0.97	42.85
17	Benzoic acid	1.48	0.05	0.64	6.81
18	Histidine	1.46	0.13	0.97	−23.75
19	Uracil	1.42	0.15	0.99	−1.08
20	Sucrose	1.41	0.04	0.73	9.17
21	Malic acid	1.39	0.10	0.89	−0.39
22	Serine	1.37	0.10	0.93	−5.55
23	Threitol	1.36	0.05	0.71	−1.11
24	Phenylalanine	1.35	0.15	0.99	−39.56
25	Erythronic acid lactone	1.30	0.08	0.80	1.74
26	Leucine	1.29	0.13	0.99	−38.43
27	Adenosine-5-phosphate	1.27	0.12	0.97	−4.98
28	Palmitic acid	1.23	−0.13	−0.79	25.12
29	3-Phosphoglycerate	1.22	−0.12	−0.99	297.76
30	Fructose-6-phosphate	1.21	0.13	0.99	−21.05
31	Inositol-4-monophosphate	1.21	0.12	0.99	−37.26
32	Alanine	1.19	0.13	0.99	−27.64
33	Pyrophosphate	1.13	−0.12	−0.99	41.90
34	Gluconic acid	1.09	0.08	0.93	−35.19
35	Asparagine	1.09	0.12	0.99	−3.23
36	Pentadecanoic acid	1.07	−0.10	−0.96	32.98
37	Tyrosine	1.02	0.10	0.97	1.73
38	Threonine	1.00	−0.08	−0.94	23.50
39	Cellobiose	1.00	0.10	0.94	8.64

VIP: variable influence on the projection; *p*[1]: the loading of the first predictive component to explain the largest variation; *p*(corr)[1]: the correlation coefficient between the metabolite and the score of the first predictive component to explain the largest variation; P: phosphate.
